# B_12_X_11_(H_2_)^−^: exploring the limits of isotopologue selectivity of hydrogen adsorption†

**DOI:** 10.1039/d1ra06322g

**Published:** 2021-09-16

**Authors:** Toshiki Wulf, Jonas Warneke, Thomas Heine

**Affiliations:** Wilhelm Ostwald Institute of Physical and Theoretical Chemistry, Leipzig University Linnéstr. 2 04103 Leipzig Germany toshiki.wulf@uni-leipzig.de; Institute of Resource Ecology, Research Site Leipzig, Helmholtz-Zentrum Dresden-Rossendorf Permoserstr. 15 04318 Leipzig Germany thomas.heine@tu-dresden.de; Leibniz Institute of Surface Engineering (IOM) Permoserstr. 15 04318 Leipzig Germany; Faculty of Chemistry and Food Chemistry, School of Science, TU Dresden 01062 Dresden Germany

## Abstract

We study the isotopologue-selective binding of dihydrogen at the undercoordinated boron site of B_12_X_11_^−^ (X = H, F, Cl, Br, I, CN) using *ab initio* quantum chemistry. With a Gibbs free energy of H_2_ attachment reaching up to 80 kJ mol^−1^ (Δ*G* at 300 K for X = CN), these sites are even more attractive than most undercoordinated metal centers studied so far. We thus believe that they can serve as an edge case close to the upper limit of isotopologue-selective H_2_ adsorption sites. Differences of the zero-point energy of attachment average 5.0 kJ mol^−1^ between D_2_ and H_2_ and 2.7 kJ mol^−1^ between HD and H_2_, resulting in hypothetical isotopologue selectivities as high as 2.0 and 1.5, respectively, even at 300 K. Interestingly, even though attachment energies vary substantially according to the chemical nature of X, isotopologue selectivities remain very similar. We find that the H–H activation is so strong that it likely results in the instantaneous heterolytic dissociation of H_2_ in all cases (except, possibly, for X = H), highlighting the extremely electrophilic nature of B_12_X_11_^−^ despite its negative charge. Unfortunately, this high reactivity also makes B_12_X_11_^−^ unsuitable for practical application in the field of dihydrogen isotopologue separation. Thus, this example stresses the two-edged nature of strong H_2_ affinity, yielding a higher isotopologue selectivity on the one hand but risking dissociation on the other, and helps define a window of adsorption energies into which a material for selective adsorption near room temperature should ideally fall.

## Abstract 2

The extreme H_2_ affinity of B_12_X_11_^−^ gives a glimpse of how higher selectivities in adsorptive isotopologue separation may be achieved.

## Graphical abstract

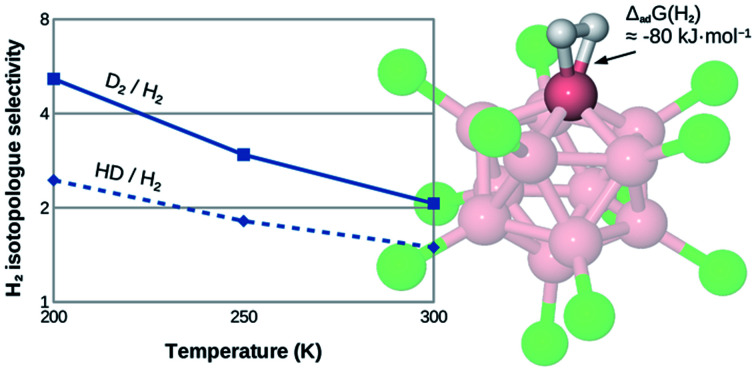

## Introduction

As the element with the lowest mass, hydrogen has the most pronounced isotope effect of all elements. This leads to numerous applications of heavy hydrogen isotopes, deuterium (^2^H = D) and tritium (^3^H = T): in spectroscopy; structural analysis and the elucidation of reaction mechanisms, especially in biochemistry and pharmaceutical research;^1,2^ and – more recently – even in pharmaceuticals,^3,4^ where an effort is ongoing to exploit the slower rate of reaction of deuterated compounds to delay or alter metabolic pathways in order to increase potency and/or reduce side effects.

The continuous need for heavy hydrogen for energy production, science and medicine has motivated a revision of state-of-the-art separation processes^5^ by employing nanostructured materials. In a relatively recent approach, penetration and tunneling through two-dimensional materials such as graphene or hexagonal boron nitride are used to separate hydrogen isotopes.^6,7^ Other separation techniques involve kinetic sieving in the interstitial space of layered materials^8^ or in apertures in metal–organic frameworks (MOFs),^9^ or isotopologue-selective adsorption (chemical affinity quantum sieving, CAQS)^10^ at strong metal sites in MOFs^11–13^ and zeolites.^14,15^

CAQS relies on the different zero-point energies of the H_2_ isotopologues, which approximately correlate with the square root of the mass of the isotopologue. This leads to a stronger adsorption of the heavier isotopes and results in higher selectivities when the zero-point energy of adsorption is higher. The adsorption energy itself also positively correlates with the selectivity (albeit more loosely), because a higher adsorption energy leads to a steeper potential energy surface, which in turn increases the zero-point energy. (Although the stretching of the H–H bond decreases the frequency of the corresponding vibrational mode and thereby the zero-point energy, this effect is overcompensated by the contributions from other modes.^16,17^).

Recent progress has been made to enable CAQS significantly above the boiling temperature of liquid nitrogen. For example, Cu(i)-MFU-4*l* with an H_2_ adsorption enthalpy on the order of 30–35 kJ mol^−1^ and a zero-point energy difference of 2.5–3.0 kJ mol^−1^ between D_2_ and H_2_ reaches a D_2_/H_2_ selectivity of 10 at 100 K. However, since both selectivity and uptake decline with increasing temperature, operating temperatures must remain well below 200 K.^12^ Nevertheless, these experiments raised hopes that an appreciable selectivity at or near room temperature could be achieved if materials or adsorption sites with higher adsorption energies could be found. With this publication, we want to make a contribution towards the search of such materials.

B_12_X_11_^−^ is a fascinating ion. Derived from the highly stable^18–20^ dianion B_12_X_12_^2−^ by abstraction of X^−^, its reactivity is driven by the strong preference for a double negative charge.^21,22^ Despite its negative charge, the monoanion is therefore highly electron-deficient. The partial positive charge at the undercoordinated boron atom would be expected to lead to strong H_2_ attraction in a way similar to undercoordinated metal cations.^17^ The significant interaction between the undercoordinated boron site and noble gas atoms reported by experiment and theory^21–24^ suggests that this system will also strongly bind dihydrogen, and that gas-phase experiments addressing this interaction should be feasible. A particularly attractive feature of these systems is the prospect of modifying the substituents X in order to form a cavity that sterically confines the bound dihydrogen to further increase isotopologue selectivity. For these reasons, we consider B_12_X_11_^−^ a suitable model system to study the limits of isotopologue-selective H_2_ adsorption.

In this article, we computationally study H_2_ attachment at B_12_X_11_^−^ (X = H, F, Cl, Br, I, CN). Using *ab initio* methods, we calculate thermodynamic parameters of the attachment reaction, analyze the influence of X on these parameters and of the different vibrational modes on the isotopologue selectivity. Furthermore, we investigate entropy effects, calculate isotopologue selectivities of the hypothetical adsorption of molecular H_2_ and examine potential dissociation pathways for H_2_ bound at B_12_X_11_^−^. Therefore, the present study highlights a wide range of aspects that are useful for the rational design of materials for dihydrogen isotopologue separation, including some rather surprising findings on the relation of attachment energy and selectivity, parameters that can be adjusted to enhance selectivity, and obstacles such as dihydrogen dissociation.

## Methods

B_12_X_11_^−^ (with X = H, F, Cl, Br, I and CN) and their H_2_ adducts have been optimized with hybrid density functional theory using the PBE0 (ref. 25) functional. Dispersion interactions have been accounted for with Grimme's D3 approach and Becke–Johnson damping, commonly referred to as D3(BJ).^26^ Equilibrium structures, their energies and Hirshfeld charges have been obtained using geometry optimization employing Ahlrich's def2-QZVP split-valence quadruple-zeta basis set.^27^ Potential energy surface scans have been performed using the smaller def2-TZVP basis set from the same series. For all DFT calculations we have made use of the resolution-of-identity and chain-of-spheres approximations, RIJCOSX,^28^ in conjunction with the def2/J^29^ auxiliary basis.

Thermal and zero-point energy contributions to enthalpies and entropies have been calculated at the above-mentioned DFT level using numerical harmonic vibrational frequency analysis. Standard increments of 0.005 Bohr have been used except for B_12_H_11_(H_2_)^−^ and B_12_F_11_(H_2_)^−^ where this led to imaginary frequencies; 0.002 and 0.001 Bohr, respectively, have been used in those cases to ensure consistent treatment in thermodynamic calculations by ORCA. The Gibbs free energy has been calculated using the quasi-rigid-rotor-harmonic-oscillator (QRRHO) approximation^30^ as implemented in ORCA. This approximation treats the entropy (but not internal energy) contributions of low-frequency vibrational modes as free internal rotations by default. In line with such treatment, the zero-point and thermal vibrational contributions of the internal rotation to the internal energy have been subtracted from the value calculated by ORCA and ½*RT* has been added instead. Finally, entropy contributions have been corrected for an external symmetry number of 5 for B_12_X_11_^−^ and an internal symmetry number for B_12_X_11_(H_2_)^−^: 10 for homoisotopic cases and 5 for heteroisotopic ones.^31^ For the B_12_X_11_(H_2_)^−^ species, the Gibbs energies have been calculated for both symmetrical orientations of H_2_ with respect to B_12_X_11_^−^ and the average has been used subsequently.

The hypothetical separation factors between two isotopologues of H_2_ (below referred to as *i* and *j*) have been calculated from the Gibbs energies of attachment of H_2_ (for the reaction leading to the local minima with undissociated H_2_) *via* the respective equilibrium constants:
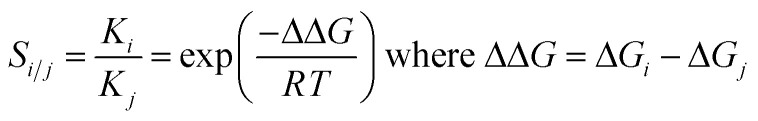


Correlated single-point calculations have been performed using the DLPNO-CCSD(T)^32,33^ method as implemented in ORCA in conjunction with the def2-QZVPP and def2-QZVPP/C^34^ basis sets based on a Hartree–Fock calculation with VeryTightSCF settings. SCF convergence issues, which arose in some cases, have been cured using the SlowConv setting and employing the SOSCF algorithm late in the SCF procedure (SOSCFStart 0.0001). For X = H, NormalPNO and TightPNO^35^ settings have been compared and found to give almost identical results; NormalPNO has therefore been used throughout. Similarly, def2-TZVPP, def2-QZVPP, aug-cc-pVTZ and aug-cc-pVQZ basis sets have been found to yield very similar reaction energies for X = H, which has been taken as justification for using def2-QZVPP results without further refinement or correction for basis set effects.

Calculations employed ORCA^36^ version 4.2.1, except for energy decomposition analysis (EDA). The latter calculations have been performed with the Amsterdam Density Functional (ADF) program from the Amsterdam Modeling Suite (AMS), version 2019.104 (r76620),^37,38^ performing single-point calculations at the PBE0-D3(BJ) level in conjunction with scalar ZORA^39^ for relativistic effects and the TZ2P^40^ basis set with native Slater-type orbitals.

## Results and discussion

### Structure of B_12_X_11_^−^ and B_12_X_11_(H_2_)^−^

The structure of B_12_X_11_^−^ (X = F, Cl, Br, I, CN) with and without attached noble gas atoms has been computationally investigated several times^21–23^ and discussed in detail recently:^24^ Compared to B_12_X_12_^2−^, the undercoordinated boron atom (B^0^) in B_12_X_11_^−^ moves toward the center of the cluster. Meanwhile, the substituents X of the surrounding boron atoms slightly bend towards B^0^, thereby decreasing the angle *α*(X–Ctr–B^0^) between X, the center of the cluster (Ctr) and B^0^ from 63.4° to 60.5°. Upon binding of noble gas atoms, this angle remains practically unchanged.

Given the similar size of He, Ne and H_2_, one might expect B_12_X_11_(H_2_)^−^ to behave similarly to B_12_X_11_He^−^ and B_12_X_11_Ne^−^. However, the latter two complexes show distinct differences in their B^0^–noble gas bond length. Moreover, H_2_ has a much higher HOMO, lower LUMO and is more polarizable. Therefore, it should bind much more strongly to B_12_X_11_^−^. This expectation is reflected in the B^0^–H_2_ distances, which are much shorter than those of B^0^–He despite similar sizes.

**Fig. 1:**
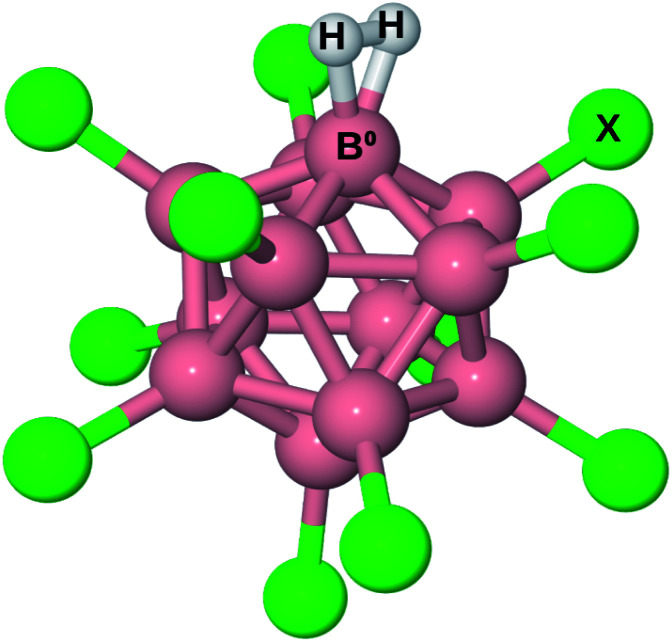
Illustration of B_12_X_11_^−^ with H_2_ attached. B^0^ is the boron atom which is undercoordinated in B_12_X_11_^−^ and to which H_2_ is bound in B_12_X_11_(H_2_)^−^.

The structure of B_12_X_11_(H_2_)^−^ is shown in Fig. 1. For all X investigated, the H–H bond is elongated by more than 9 pm, that is at least 3 pm more than for H_2_ coordinated at (C_4_H_8_O_2_)Cu^+^ (C_4_H_8_O_2_ = 1,4-dioxane; calculated value from ref. 17). A comparison of the geometric parameters, which are listed in Table 1, reveals that X = F is an outlier with a much longer H–H bond length and a shorter B^0^–H_2_ distance, which has consequences for the thermodynamic properties and isotopologue selectivity as discussed below.

**Table 1:** Characteristic geometric parameters of B_12_X_11_(H_2_)^−^: H–H bond length (minimum on the potential energy surface at the PBE0-D3(BJ) level) and distance between B^0^ and center of H_2_. All distances in pm

X	*r*(H–H)	*R*(B^0^–H_2_)
(Free H_2_)	74.4	
H	85.0	125.7
F	91.0	119.6
Cl	85.2	125.2
Br	84.5	126.4
I	84.1	127.6
CN	84.2	127.3

### Analysis of the dihydrogen–B_12_X_11_^−^ interaction

**Fig. 2:**
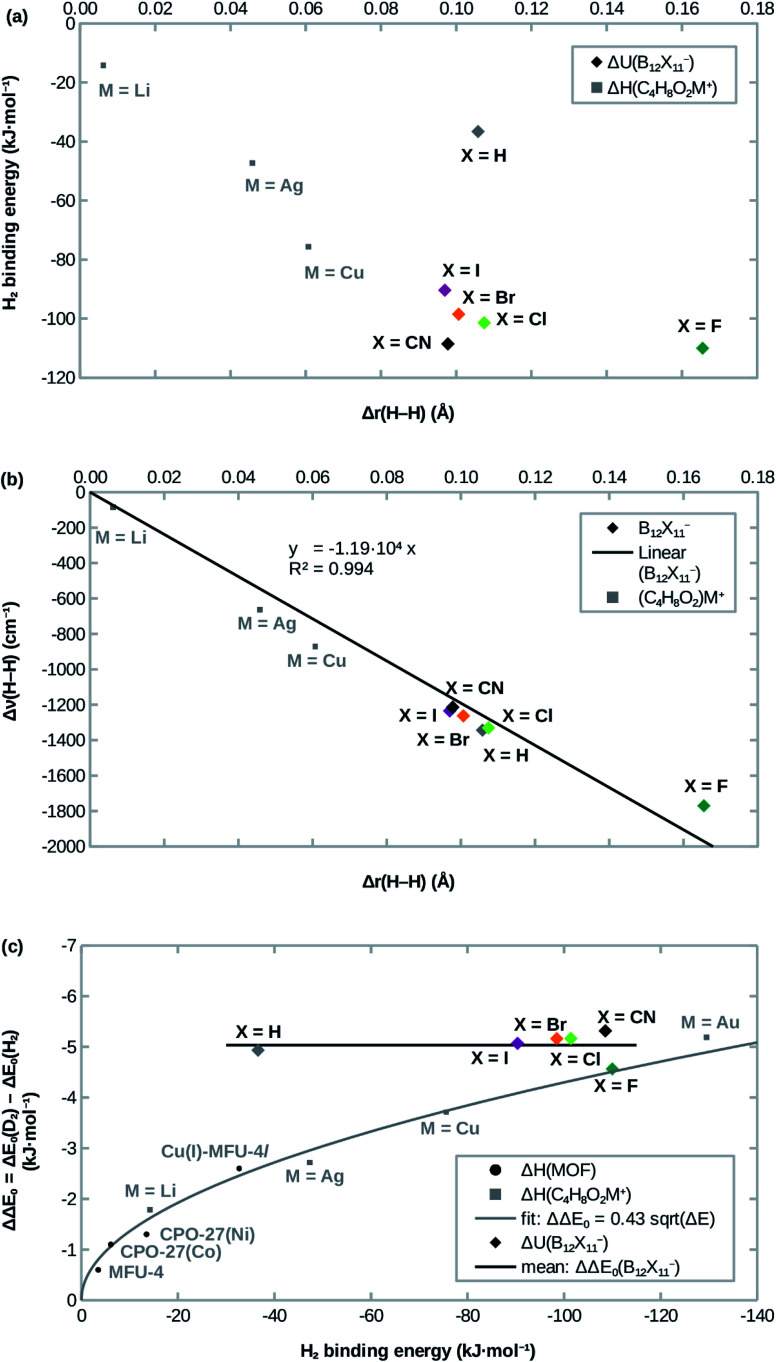
Correlation of (a) the H_2_ attachment energy and (b) the red-shift of H–H stretch frequency Δ*ν*(H–H) with the H–H bond length elongation Δ*r*(H–H) w.r.t. free H_2_ in B_12_X_11_^−^ and dioxane complexes. (c): Comparison of H_2_ attachment energies and differences of zero-point energies of attachment ΔΔ*E*_0_ between D_2_ and H_2_. Experimental values for MOFs are from ref. 12,13,41, calculated values for dioxane complexes (C_4_H_8_O_2_M^+^) are from ref. 17.

Attachment energies for H_2_ coordinating side-on at B_12_X_11_^−^ are shown in Table 2 and have, with the exception of X = H, a magnitude of ≈100 kJ mol^−1^, which is much stronger than for most interactions of H_2_ with undercoordinated metal sites.^11,12^ As would be expected from the significantly elongated H–H bonds, the title compounds are predicted to strongly attract H_2_. However, the order CN ≈ F > Cl ≳ Br > I ≫ H from strongest to weakest interaction differs from what would be expected from the bond lengths (Table 1). This surprising lack of correlation, which is shown in Fig. 2a, is contrary to systems where undercoordinated metal sites serve as attractors and where bond length and binding energy are evidently correlated.

**Table 2:** Internal energies of H_2_ and D_2_ attachment at B_12_X_11_^−^ calculated for 0 K, difference of this energy between D_2_ and H_2_ (which is equivalent to the difference in zero-point energy), Gibbs energies of attachment for 300 K, its difference between D_2_ and H_2_ and the resulting D_2_/H_2_ selectivity at 300 K. All energies are in kJ mol^−1^; selectivities in units of one

X =	Δ_ad_*U* (H_2_)	Δ_ad_*U* (D_2_)	ΔΔ*E*_0_	Δ_ad_*G* (H_2_)	Δ_ad_*G* (D_2_)	ΔΔ*G*	*S* (D_2_/H_2_)
H	−36.6	−41.5	−4.9	−9.1	−10.8	−1.72	1.99
F	−110.0	−114.6	−4.6	−81.3	−82.6	−1.31	1.69
Cl	−101.4	−106.6	−5.2	−72.5	−74.3	−1.81	2.06
Br	−98.5	−103.6	−5.2	−70.1	−71.9	−1.77	2.04
I	−90.4	−95.4	−5.1	−62.0	−63.7	−1.67	1.96
CN	−108.5	−113.9	−5.3	−80.0	−81.8	−1.87	2.12

Interestingly, the H–H bond is much more elongated for X = F compared to other X, despite a binding energy which is only slightly higher. Conversely, X = H shows a substantially lower binding energy (36 kJ mol ^−1^*vs.* > 90 kJ mol ^−1^) despite geometric parameters which are very similar to X = Cl. Furthermore, X = CN, which has the second-highest H_2_ binding energy after X = F, lies in between the least strongly binding halogens X = Br and X = I in terms of the H–H bond length. Only for X = Cl, Br, I does the binding energy follow the trend expected from the DFT-predicted bond lengths, that is: among the three, B_12_Cl_11_(H_2_)^−^ has the highest binding energy, the shortest B^0^–H_2_ distance and the most elongated H–H bond, followed by B_12_Br_11_(H_2_)^−^ with B_12_I_11_(H_2_)^−^ being last.

If the interaction between H_2_ and all investigated B_12_X_11_^−^ was mainly driven by σ bonding (from H_2_ to B^0^), one would expect a strong correlation between the charge at B^0^ and the H_2_ attachment energy. At least for Cl, Br and I, a reasonable correlation between attachment energy and Hirshfeld charge at B^0^ is found (see ESI; Fig. S1†). However, the correlation is poor for other X and different charge trends are obtained when using orbital-based charges such as NPA charges (Fig. S2†). Furthermore, this does not explain the significant elongation of the H–H bond for X = F.

Since these unintuitive findings warrant a deeper investigation of the underlying binding properties, we have performed energy decomposition analysis (EDA) of the Gibbs energy of the attachment reaction of H_2_ at B_12_X_11_^−^. Results are shown in Table 3: With one exception (Δ*E*_def_ of B_12_X_11_^−^, for discussion see below), the contributions of dispersion interaction and geometry deformation of the reactants as well as zero-point and thermal contributions are very similar.

**Table 3:** Contributions to the Gibbs free energy of attachment (300 K) of H_2_ at B_12_X_11_^−^ from energy decomposition analysis (EDA): Pauli repulsion energy Δ*E*_Pauli_, electrostatic interaction (including nuclear repulsion) Δ*E*_elstat_, orbital interaction Δ*E*_oi_, explicit dispersion correction Δ*E*_disp_, geometry deformation Δ*E*_def_ of B_12_X_11_^−^ and H_2_, Born–Oppenheimer energy Δ*E*_el_ (sum of electronic and nuclear contributions, *i.e.* all of the aforementioned ones), zero-point energy Δ*E*_0_, thermal contributions Δ*E*_therm_ at 300 K (mostly entropy) and total Gibbs energy Δ*G* (sum of Δ*E*_el_, Δ*E*_0_ and Δ*E*_therm_). Δ*G* values differ from Table 2 because EDA is at the DFT level; values in Table 2 are expected to be more accurate. All energies in kJ mol ^−1^

X =	Δ*E*_Pauli_	Δ*E*_elstat_	Δ*E*_oi_	Δ*E*_disp_	Δ*E*_def_ (B_12_X_11_^−^)	Δ*E*_def_ (H_2_)	Δ*E*_el_	Δ*E*_0_	Δ*E*_therm_	Δ*G*
CN	400	−166	−402	−10	13	19	−146	22	29	−95
Cl	435	−185	−405	−10	16	16	−134	22	29	−84
Br	444	−185	−406	−11	14	14	−130	21	28	−81
I	476	−192	−420	−13	13	13	−122	21	28	−73
H	**490**	−205	**−385**	−7	15	19	−72	21	27	−23
F	480	−213	**−462**	−7	**34**	18	−150	23	29	−99

The greatest variability is afforded by the Pauli repulsion term, which results from the antisymmetry requirement of the wave function and closely matches the intuitive understanding of steric repulsion. For CN, Cl, Br and I, Pauli repulsion is the dominating contributor to the overall trend in the reaction Gibbs energies. It is partly compensated by the electrostatic interaction, which may sound surprising at first, but is justified given the well-known fact^42^ that molecules attract each other more strongly the more diffuse their charge clouds are.

EDA shows two main reasons for the B_12_X_11_^−^–H_2_ interaction being weakest for X = H. One is the weaker orbital interaction. The other is the stronger Pauli repulsion, which is caused by the larger electron density at B^0^ due to the less electron-withdrawing nature of X = H (see also ESI; Fig. S3†).

Finally, the case of X = F is characterized by a substantially stronger orbital interaction with H_2_. This is unlike what has been observed for the attraction of noble gas atoms, which is weakest for X = F. This striking difference is in line with results from a recent publication,^43^ which compared the binding of noble gases, N_2_ and CO at B_12_X_11_^−^. It was found that X = F shows the weakest binding of noble gas atoms but the strongest binding of N_2_ and CO due to π-like backbonding from B^0^ into the antibonding σ* orbitals of the attached molecule (something which is not possible for noble gases). This binding of N_2_ and CO for X = F is unlike the strong binding for X = CN, which is based almost solely on strong σ bonding, a binding mode which is much weaker for X = F. We expect similar behavior for the binding of H_2_, *i.e.* strong σ bonding and weak backbonding with modest H–H elongation for X = CN (and to a lesser degree Cl, Br, I) and weaker σ bonding but strong backbonding for X = F. The stronger backbonding would also explain the much greater elongation of the H–H bond for X = F.

### Very similar zero-point energies of attachment

Previous studies of undercoordinated metal sites show a substantial (albeit sublinear) increase of the adsorption zero-point energy with the adsorption energy in paddlewheel MOFs^16^ and zeolites^17^ (see also Fig. 2c). By contrast, all B_12_X_11_^−^ investigated here have very similar zero-point energies of attachment, which are almost independent of X (see Fig. 2c).

In the case of X ≠ H, this could be interpreted as entering a region of saturation where an increase in the attachment energy yields smaller and smaller gains in the selectivity. However, this does not explain the case of X = H, where a lower zero-point energy of attachment would have been expected.

**Fig. 3:**
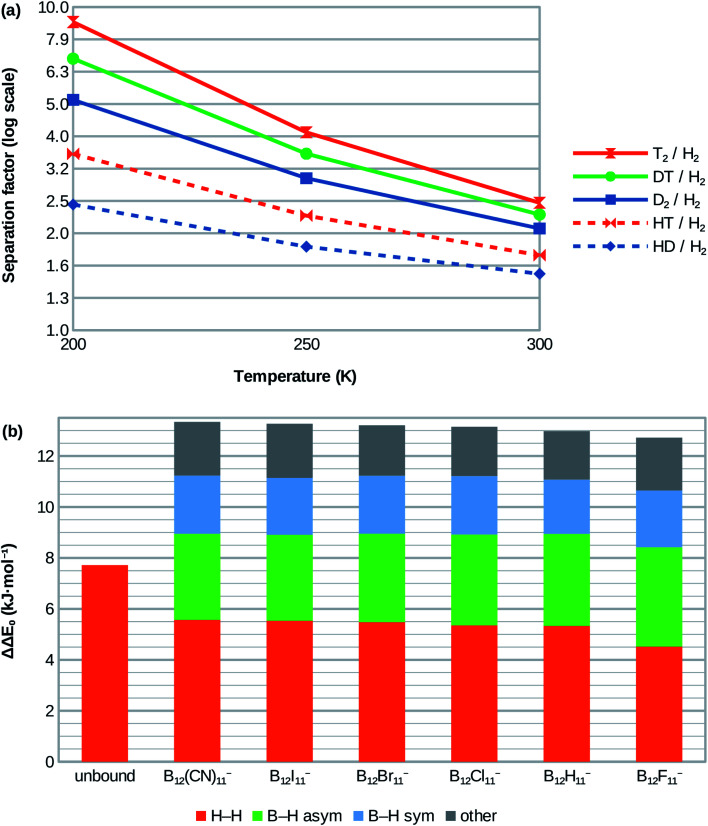
(a) Calculated isotopologue selectivities for the H_2_ attachment at B_12_X_11_^−^ using X = Cl as example. Very similar selectivities are obtained for X = H, Br, I, CN and lower ones for X = F (see ESI; Table S1†). (b) Contribution of vibrational modes to zero-point energy difference between D_2_ and H_2_ isotopologues for free (unbound) dihydrogen in the gas phase (left bar) and when coordinated at the various B_12_X_11_^−^.

An explanation is given by the ZPE contributions of the different vibrational modes (see below for a detailed discussion). The greatest ZPE variability is afforded by the asymmetrical B^0^–H stretch mode, which works in favor of the overall selectivity. However, most of this contribution is compensated by the weakening of the H–H bond with an opposite contribution (see Fig. 3b).

### Vibrational modes in detail

When discussing the vibrational modes of B_12_X_11_^−^, the internal rotation (*φ* in previous publications^12,17^) of H_2_ about the B^0^–H_2_ axis is of some interest. The combination of five-fold symmetry of B_12_X_11_^−^ and two-fold symmetry of H_2_ results in a ten-fold internal rotational symmetry (36° period) about the B^0^–H_2_ axis^31^ for homoisotopic H_2_ (five-fold internal rotational symmetry for heteroisotopic H_2_). The high symmetry leads to an exceptionally flat potential energy surface for this mode, which is confirmed by a scan of the potential energy surface using DFT: the obtained differences between highest and lowest energy are below 0.02 kJ mol^−1^ in all cases, which is indistinguishable from numerical noise (see NEB paths for the internal rotation in the ESI†). A comparison of the vibrational behavior of B_12_X_11_(H_2_)^−^ for *φ* = 0° and *φ* = 90° (equivalent to *φ* = 18°) shows almost identical results for the frequencies of the other modes (differences of less than 5 cm^−1^). These small differences suggest that the coupling between the quasi-free rotational mode along *φ* and the remaining vibrational modes is negligible. This allows for treatment of the other modes independent of *φ* and thereby greatly simplifies the calculation of the ZPE and of the thermal contributions to the Gibbs energies.

H_2_ coordinated at B_12_X_11_^−^ exhibits a vibrational behavior that qualitatively matches that observed in previous studies of highly attractive metal centers.^12,16^ The highest-frequency mode is the H–H stretch vibration (*ν*_HH_ along *r*), which is strongly red-shifted with respect to the free molecule. It is followed by the asymmetrical B^0^–H stretch vibration (*ν*_BH,*asym*_, which in the case of weaker interaction would have been denoted as rotation of H_2_ in the B^0^–H_2_ plane along the angle *ϑ*) and the (symmetrical) stretch vibration of the B^0^–H_2_ bond (*ν*_BH,sym_ along *R*).

As shown in Fig. 2b, the frequency shift of the H–H stretch vibration correlates almost linearly with the H–H distance, not only for different B_12_X_11_^−^, but also when expanding the dataset using data from different (C_4_H_8_O_2_)M^+^ calculated in ref. 17. Near-linearity holds true even across different classes of compounds and breaks down only at extreme elongations (H_2_ at B_12_F_11_^−^ and C_4_H_8_O_2_Au^+^; the latter is not shown in Fig. 2b because of the extreme values of Δ*r* = 100 pm and Δ*ν* = 3722 cm^−1^). These cases, where the H–H stretch frequency approaches zero and the H–H distance goes to infinity, however, are still very well described by Badger's rule,^44^ which implies *ν* ∝ *r*^−3/2^ for H_2_.

Likewise, it could be expected that the frequencies of the B^0^–H stretching modes correlate with the B^0^–H_2_ distance. This is indeed the case for *ν*_BH,asym_, but surprisingly not for *ν*_BH,sym_.

The two remaining degrees of freedom, which can be interpreted as the hindered translations of H_2_ perpendicular to the B^0^–H_2_ axis, are expected to correlate more with the degree of steric hindrance than with the attractiveness of the undercoordinated site itself. Unfortunately, the substituents X are too far away from the H_2_ binding site to exert any significant influence leading to little variation in the frequencies of these modes.

### Zero-point energies of attachment, entropies and high predicted D_2_/H_2_ selectivities

Although H_2_ attachment energies at B_12_X_11_^−^ vary widely with X, the differences between the Gibbs energies of attachment of H_2_ and D_2_ for a given X and therefore the selectivities are in a very narrow range (with X = F being a slight outlier). This may be surprising at the face of it, but is in line with the very similar zero-point energies of attachment of all species.

The entropy of H_2_ attachment is negative because the translational and rotational entropies of the free H_2_ are higher than the vibrational entropy of the bound H_2_, an effect which is more pronounced the higher the temperature. This fight against entropy is one of the key obstacles towards adsorptive H_2_ isotopologue sieving at high temperatures. Therefore, the high predicted D_2_/H_2_ selectivity – which is approximately 2.0 at 300 K for X = H, Cl, Br, I, CN – is truly remarkable. Selectivities of about 2.9 and 5.1 are predicted for 250 K and 200 K, respectively. Note, that the selectivities at 250 K and 200 K are much higher than would be expected based on the room temperature value if only the direct influence of temperature *T* on the separation factor *via α* = exp(-ΔΔ*G*/*RT*) was considered. This is because most of the selectivity loss at higher temperatures is due to the above-mentioned entropy effects, which result in lower absolute values of ΔΔ*G* as temperature increases and therefore lower selectivities.

HD/H_2_ selectivities are predicted to be about 1.4 at 300 K, 1.8 at 250 K and 2.4 at 200 K. Even at 200 K, T_2_/D_2_ and DT/D_2_ selectivities are much lower at around 1.7 and 1.3, respectively. More information is given in Fig. 3a and in the ESI (Table S1†).

### Heterolytic dissociation of H_2_ at B_12_X_11_(H_2_)^−^

**Fig. 4:**
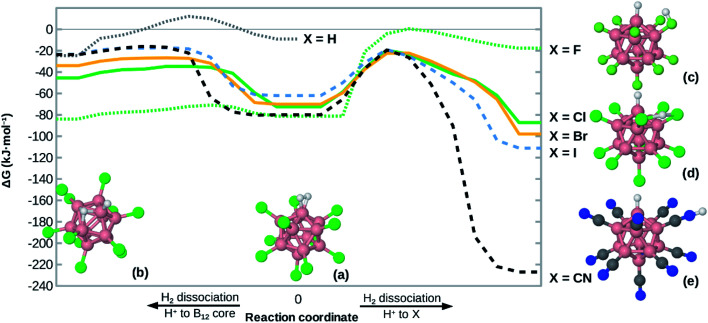
Potential energy surface of H_2_ bound at B_12_X_11_^−^. The (local) minima in the middle (a) correspond to undissociated H_2_, the other ones (b–e) to heterolytically dissociated H_2_ (with H^−^ at B^0^). The minima on the left (b) have H^+^ bound at the B_12_ cage. For the minima on the right, H^+^ either binds to one X – which is a local minimum for X = F (c) and X = CN (e) – or between two X, which is a local minimum for X = Cl, Br, I (d). The curve for X = H does not show the right side as structures (a) and (c) would be equivalent in that case. Except for X = F, the two transition states shown are similar not only in energy but also in structure. The zero level of the energy corresponds to the unbound state (infinite distance between H_2_ and B_12_X_11_^−^). Energies for local minima are given at the DLPNO-CCSD(T)//PBE0-D3(BJ) level, other points are interpolated using PBE0-D3(BJ) data. Colors: B pinkish, H white, X green, C gray, N blue.

Given the high H_2_ binding energies, one might expect that the title compounds show a substantial propensity towards dissociation of coordinated H_2_. Indeed, we found local minimum structures with a negatively polarized H^−^ at B^0^ and a positively polarized H^+^. As shown in Fig. 4, that H^+^ can coordinate at one X (for X = F, CN), between two X (for X = Cl, Br, I) or directly at the boron core (preferred only for X = H, F), behavior which is in line with observations on B_12_X_12_H^−^ (X = F, Cl, Br, I)^45^ and B_12_X_11_RH^−^ (R = alkyl group).^46^

The dissociation behavior of H_2_ at B_12_X_11_^−^ is, however, unlike what has been observed in the case of undercoordinated metal sites. In a previous publication we have predicted particularly strong H_2_ interaction at zeolitic Au^+^ sites^17^ and observed strong elongation of the H–H bond to the point of dissociation using local coupled-cluster calculations. However, dissociation was homolytic in that case and attempts to create structures with H^+^/H^−^ polarization failed (geometry optimization reverted to structures with equivalent H atoms). This shows the uniquely strong ability of B_12_X_11_^−^ to polarize H_2_ and stabilize a H^−^ ion at the B^0^ site.

For H_2_ bound at B_12_X_11_^−^, the activation energy for heterolytic dissociation is below (or only slightly above in the case of X = H) the H_2_ attachment energy, which means that an incoming H_2_ would have enough energy to overcome the barrier and dissociate. We therefore expect that B_12_X_11_(H_2_)^−^ (X = F, Cl, Br, I, CN) will not be observed in experiment and a technological application requiring multiple adsorption cycles is not realistic. As indicated by its potential energy surface, B_12_H_11_(H_2_)^−^ may be somewhat metastable against dissociation and it could therefore be interesting to study the dynamic behavior of this ion at low temperatures. Harmonic values of characteristic vibrational frequencies are given in the ESI (Table S2†).

Compared to other X, the potential energy surface for X = F is much flatter towards H_2_ dissociation and the formation of the structure shown in Fig. 4(b), which is in line with the much longer H–H bond length in this case.

It is interesting to note that the heterolytic dissociation of heteronuclear H_2_ isotopologues (*i.e.* HD, HT and DT) shows a preference for the heavier nucleus to occupy the position where the vibrational frequency of the corresponding stretch frequency is higher, thereby minimizing the overall zero-point energy. Especially for B_12_X_11_HD^−^ with X = Cl, Br and I, this results in a pronounced preference for D to occupy the H^−^ position (coordinated at B^0^ as D^−^) and for H to take the H^+^ position between two X as the latter is characterized by a very shallow potential energy surface. Equilibrium constants are given in Table S3.†

### Related species

Preliminary calculations on related species (B_12_X_11_^2−^, B_12_X_10_^2−^ and B_12_X_10_^−^) showed a similar propensity towards dissociative binding of H_2_, albeit with even lower dissociation barriers. Given the added computational expense of treating radical systems and/or multiple isomers, they have not been studied in detail.

For the octahedral B_6_X_5_^−^, no local minimum structure with undissociated H_2_ has been found at all. Their smaller structures lead to smaller B–B–B angles, stronger pre-hybridization and therefore much stronger interaction.

## Conclusion

We have investigated the interaction of B_12_X_11_^−^ with H_2_ with a special focus on the different interaction strengths of H_2_ and D_2_. Internal energies of attachment are highest for X = CN and X = F (Δ*U* (^1^H_2_) = −110 kJ mol^−1^ at 0 K), decrease with increasing mass for the halogens (down to −90 kJ mol^−1^ for X = I) and are lowest for X = H (−36 kJ mol^−1^). Despite widely varying attachment energies, zero-point energy differences between D_2_ and H_2_ attachment are very similar at ≈5.0 kJ mol^−1^ (in contrast to results from other materials where a positive correlation between both parameters was typically found). Gibbs energies for different isotopologues are reported as well, corresponding to D_2_/H_2_ selectivities of ≈2.0 at 300 K.

Unfortunately, the strong H_2_ activation leads to heterolytic dissociation with activation energies below the interaction energy; that is, H_2_ approaching the cluster has enough kinetic energy to overcome the activation barrier and form a compound with dissociated H_2_. This highlights the very high electrophilicity of B_12_X_11_^−^ despite its negative charge, even exceeding that known from many strong H_2_-adsorbing metals like Au^+^ in zeolitic environments.

Although heterolytic dissociation likely precludes experimental investigation of B_12_X_11_(H_2_)^−^ (with undissociated H_2_) and practical application for hydrogen isotopologue separation, the example is instructive as it shows the limits of increased adsorption energy with regards to enhancing isotopologue selectivity: not only do higher adsorption energies run the risk of leading to H_2_ dissociation, but there also seems to be a limit to the adsorption zero-point energy between H_2_ and D_2_ of around 5 kJ mol^−1^. However, already this 5 kJ mol^−1^ difference is very encouraging as it leads to predicted D_2_/H_2_ separation factors of 2.0 at 300 K and 3.0 at 250 K, figures that might be further enhanced by steric confinement and would even be high enough to enable HD/H_2_ separation if a suitable material could be found that does not lead to H_2_ dissociation.

### Outlook

Since the smaller B_6_X_5_^−^ show much stronger H_2_ affinity than B_12_X_11_^−^, we would expect higher *closo*-borates to bind H_2_ less strongly. Therefore, it could be interesting to investigate clusters like B_16_X_15_^−^, B_32_X_31_^−^ and B_42_X_41_^−^ (which derive from polyhedra dual to C_28_, C_60_ and C_70_, respectively).^47,48^ Unfortunately, the synthesis of higher *closo*-borates is faced with huge thermodynamic and kinetic obstacles.^49^ To the best of our knowledge, none of these compounds have been synthesized to date, leaving little prospect for eventual application.

Nevertheless, we believe that the two known cases of Cu(i)-MFU-4*l* (adsorption enthalpy of ≈35 kJ mol^−1^ and appreciable selectivity around 200 K) and B_12_X_11_(H_2_)^−^ (internal energy adsorption of ≈110 kJ mol^−1^ and appreciable selectivity at 300 K for X = CN, but dissociation and saturation of zero-point energy of adsorption) give us an estimate of the window of adsorption energies within which the search for materials enabling isotopologue-selective adsorption should be focused.

## Author contributions

JW proposed to investigate the H_2_ affinity of B_12_(CN)_11_^−^. TW performed all calculations and prepared the first draft of the manuscript. The authors jointly discussed results and revised the manuscript.

## Conflicts of interest

There are no conflicts to declare.

## Supplementary Material

RA-011-D1RA06322G-s001
