# Supplementary material for: B12X11(H2)−: exploring the limits of isotopologue selectivity of hydrogen adsorption
Source: RSC Adv. 2021 Sep 16;11(46):28466–75. doi: 10.1039/d1ra06322g (PMC9038111; doi:10.1039/d1ra06322g)
Supplement: RA-011-D1RA06322G-s001 [file RA-011-D1RA06322G-s001.pdf]

Electronic Supplementary Material (ESI) for RSC Advances.
This journal is © The Royal Society of Chemistry 2021

Supporting Information – T. Wulf, J. Warneke, T. Heine, *RSC Advances*, **2021**, doi:10.1039/d1ra06322g

$B_{12}X_{11}^-$: exploring the limits of isotopologue selectivity of hydrogen adsorption

Table S1: Gibbs energy of attachment (kJ mol^{-1}) of $^1H_2$ and separation factors (unit: 1) of interest between different isotopologues.

300 K	$\Delta G(^1H_2)$	D_2 / H_2	HD / H_2	T_2 / H_2	HT / H_2	T_2 / D_2	DT / D_2	D_2 / HT
**CN**	**-80.0**	2.1	1.5	2.6	1.7	1.21	1.11	1.23
**F**	**-79.8**	1.6	1.4	1.7	1.5	1.06	1.05	1.04
**Cl**	**-72.5**	2.1	1.5	2.5	1.7	1.20	1.10	1.21
**Br**	**-70.1**	2.0	1.5	2.4	1.7	1.20	1.10	1.22
**I**	**-62.0**	2.0	1.4	2.3	1.6	1.18	1.09	1.21
**H**	**-9.1**	2.0	1.5	2.4	1.7	1.19	1.10	1.17

250 K	$\Delta G(^1H_2)$	D_2 / H_2	HD / H_2	T_2 / H_2	HT / H_2	T_2 / D_2	DT / D_2	D_2 / HT
**CN**	**-85.8**	3.1	1.8	4.3	2.3	1.41	1.20	1.34
**F**	**-85.8**	2.2	1.6	2.6	2.0	1.19	1.12	1.10
**Cl**	**-78.3**	3.0	1.8	4.1	2.3	1.39	1.19	1.31
**Br**	**-75.8**	2.9	1.8	4.0	2.2	1.38	1.19	1.32
**I**	**-67.8**	2.8	1.7	3.8	2.1	1.36	1.18	1.31
**H**	**-14.8**	2.8	1.8	3.8	2.2	1.36	1.18	1.26

200 K	$\Delta G(^1H_2)$	D_2 / H_2	HD / H_2	T_2 / H_2	HT / H_2	T_2 / D_2	DT / D_2	D_2 / HT
**CN**	**-91.5**	5.5	2.5	9.8	3.6	1.79	1.36	1.52
**F**	**-91.7**	3.6	2.1	5.2	3.0	1.45	1.24	1.20
**Cl**	**-84.0**	5.2	2.4	9.0	3.5	1.74	1.34	1.47
**Br**	**-81.4**	5.1	2.4	8.9	3.4	1.74	1.34	1.49
**I**	**-73.3**	4.8	2.3	8.2	3.3	1.70	1.32	1.47
**H**	**-20.2**	4.7	2.4	8.0	3.3	1.69	1.33	1.42

Table S2: Wavenumbers (cm^{-1}) of characteristic high-frequency vibrations for different $B_{12}X_{11}H_2^-$ species (see Figure 4 of the article) at the PBE0-D3(BJ)/def2-TZVP level.

	$B_{12}X_{11}(H_2)^-$			$(B_{12}H)X_{11}H^-$		$B_{12}X_{10}(XH)H^-$	
	H–H	B–H asym	B–H sym	B–H	B_{12}–H	B–H	X–H
**CN**	3190	1960	1400	2690	1810	2720	3800
**F**	2630	2260	1320	2650	2070	2540	3690
**Cl**	3070	2060	1410	2680	1840	2680	
**Br**	3140	2010	1400	2690	1730	2680	
**I**	3170	1950	1370	2690	1600	2690	
**H**	3060	2090	1280	2610	1760		

Table S3: Zero-point energy differences (kJ mol^{-1}), Gibbs free energies (kJ mol^{-1}) and equilibrium constants (unit: 1) for the exchange of the H nuclei in the global minimum structures of $B_{12}X_{11}H_2^-$ with heterolytically dissociated heteronuclear H_2. Energies are positive (equilibrium constants < 1) when the heavier nucleus is preferred in the positively polarized position.

	ΔE_0			ΔG(300 K)			K(300 K)		
X=	HD / DH	HT / TH	DT / TD	HD / DH	HT / TH	DT / TD	HD / DH	HT / TH	DT / TD
**CN**	**0.4**	0.5	0.2	**0.7**	0.9	0.3	**0.8**	0.7	0.9
**F**	**0.0**	0.0	0.0	**-0.1**	-0.2	-0.1	**1.1**	1.1	1.0
**Cl**	**-3.0**	-4.3	-1.3	**-2.7**	-3.9	-1.1	**3.0**	4.7	1.6
**Br**	**-3.1**	-4.4	-1.3	**-2.9**	-4.1	-1.2	**3.2**	5.1	1.6
**I**	**-3.5**	-5.0	-1.5	**-3.2**	-4.5	-1.3	**3.7**	6.2	1.7
**H**	**-0.3**	-0.3	-0.1	**-0.4**	-0.6	-0.2	**1.2**	1.3	1.1

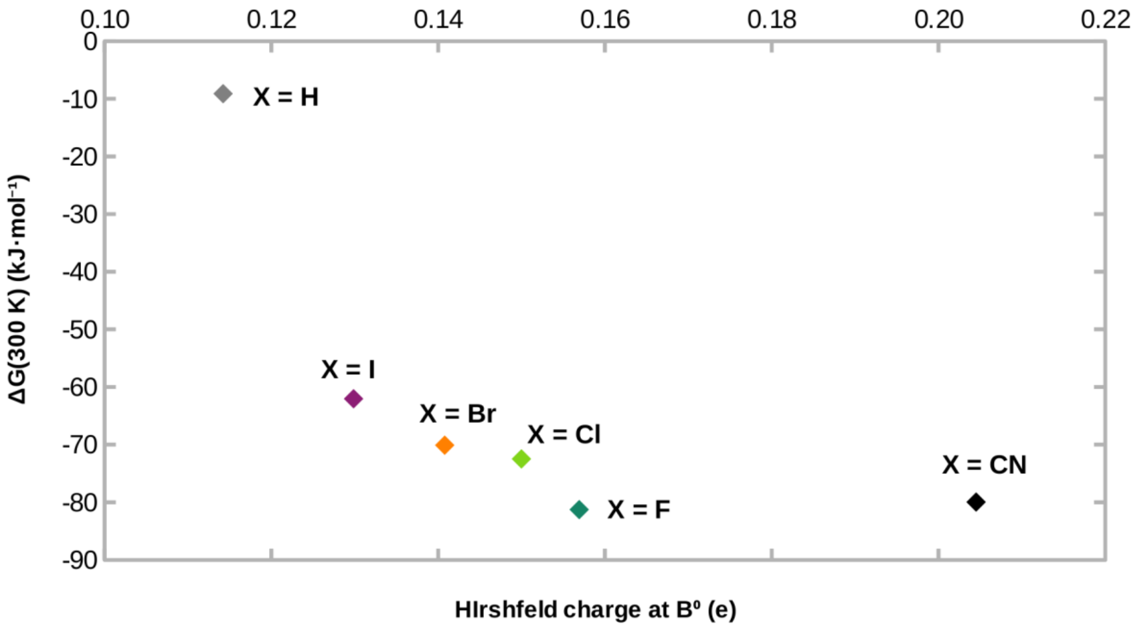

Figure S1: Gibbs free energy of attachment of H_2 at $B_{12}X_{11}^-$ and its correlation with the Hirshfeld charge.

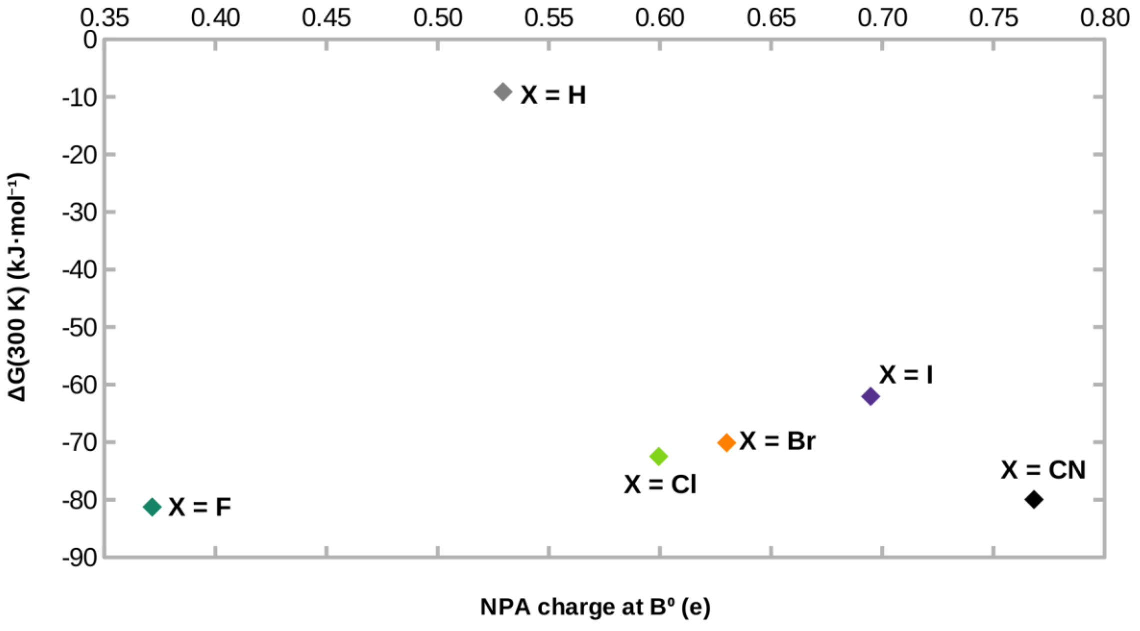

Figure S2: Gibbs free energy of attachment of H_2 at $B_{12}X_{11}^-$ and its correlation with the NPA charge.

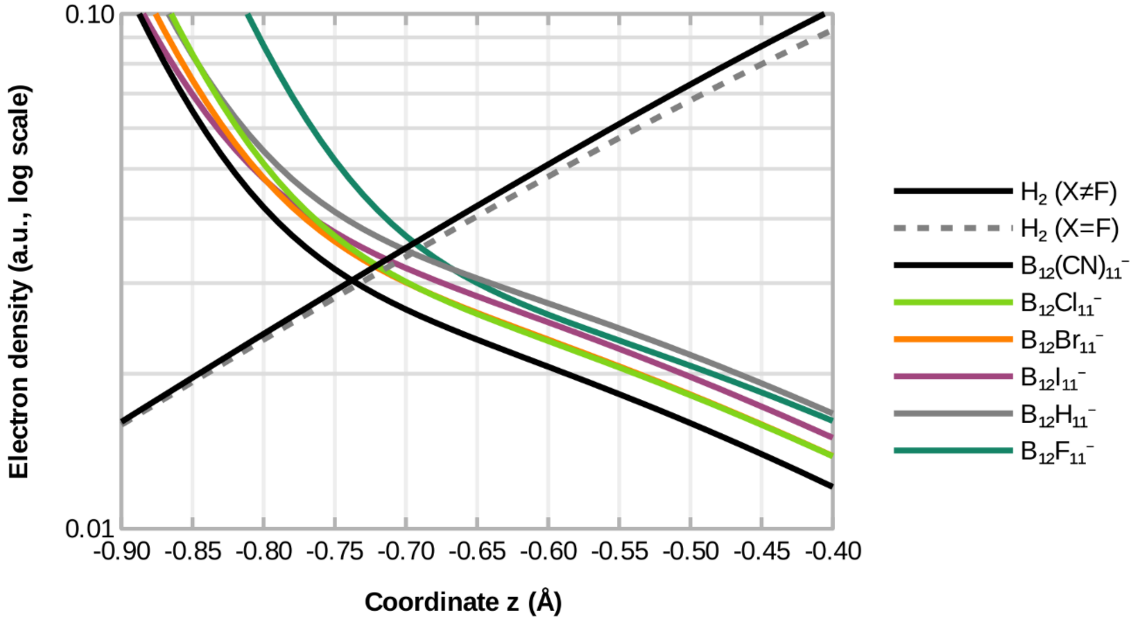

Figure S3: Electron density along z axis if the center of H_2 is placed at the origin and B^o is placed on the z axis (at approximately z = -1.25Å depending on z). Excluding X = F, the $B_{12}X_{11}^-$ electron density is consistently lowest for X = CN and highest for X = H for all distances, which is in line with the weakest and strongest Pauli repulsion, respectively. For X = F, the H–H bond is more elongated (resulting in a more delocalized and overall lower electron density), but H_2 is also much closer to B^o resulting in a different shape of the electron density with more overlap between $B_{12}X_{11}^-$ and H_2, leading to an overall high Pauli repulsion.
